# Association between lean mass, fat mass, and waist circumference with bone mineral density in Mexican children and adolescents: a cross-sectional study

**DOI:** 10.1007/s00431-025-06515-9

**Published:** 2025-10-09

**Authors:** Berenice Rivera-Paredez, Karla Muciño-Sandoval, Rafael Velázquez-Cruz, Rodolfo Rivas-Ruíz, Joacim Meneses-León, Ricardo Orozco, Carlos Esteban González-Muñoz, Juan Alfredo Tamayo-Orozco, Edgar Denova-Gutiérrez, Jorge Salmerón, Marcela Tamayo-Ortiz

**Affiliations:** 1 Research Center on Policies, Population and Health, Faculty of Medicine, National Autonomous University of Mexico, Ciudad Universitaria, Edificio CIPPS-Sótano y Piso 2, Cto. Centro Cultural S/N, Coyoacán, Mexico City, 04510 Mexico; 2 Accessalud, Mexico City, Mexico; 3 Laboratory of Genomics of Bone Metabolism, National Institute of Genomic Medicine, Mexico City, 14610 Mexico; 4 Training and Clinical Research Center, Health Research Coordination, Mexican Social Security Institute, Mexico City, 06725 Mexico; 5 Center for Evaluation and Survey Research, National Institute for Public Health, Cuernavaca, Morelos 62100 Mexico; 6 Global Mental Health Research Center, Ramón de la Fuente National Institute of Psychiatry, Mexico City, 14370 Mexico; 7 Nutrition Department, National Institute of Medical Sciences and Nutrition Salvador Zubirán, Mexico City, 14080 Mexico; 8 Department of Environmental Health Sciences, Mailman School of Public Health at Columbia University, New York, 10032 USA

**Keywords:** Childhood, Adolescents, Waist circumference, Fat mass, Lean mass, Bone mineral density

## Abstract

To assess the associations of lean mass (LM), fat mass (FM), truncal fat mass (TFM), and waist circumference (WC) with bone mineral density (BMD) in Mexican youth, independently of body weight. We analyzed cross-sectional data from 1,054 children and adolescents from the Health Workers Cohort Study. We measured total and region-specific BMD, LM, and FM, with dual-energy X-ray absorptiometry. To eliminate the effect of body weight on FM, LM, TFM, and WC, weight-adjusted values were generated using the residuals method, and then we employed multivariate linear regression models adjusted for relevant potential confounders. We further stratified the analysis by sex, age group, and sexual maturation. LM was positively associated with BMD in various anatomical sites, with β coefficients ranging from 0.004 to 0.013 in both sexes (P < 0.05). FM was inversely related to total BMD and other sites, with β coefficients ranging from -0.013 to -0.006 (P < 0.05). WC showed negative associations with BMD at some sites, with β values ranging from -0.007 to -0.002. TFM was negatively associated with BMD. During puberty, the associations were consistent across all sites, whereas this was not the case for all sites after puberty. These patterns were similarly observed across different age groups.

*Conclusion*: An increased LM and reduced FM are associated with higher BMD, particularly in the leg and hip regions, during childhood and adolescence, a critical developmental stage essential for healthy bone accrual and balance in adulthood.

**Supplementary information:**

The online version contains supplementary material available at 10.1007/s00431-025-06515-9.

## Introduction

Body composition refers to the amount and distribution of fat and fat-free tissues, including lean and bone mass, providing more specific insights than the body mass index (BMI) alone [1]. Although often attributed to aging, accumulating evidence suggests a shared pathophysiology underlying the relationship between fat, lean, and bone mass (FM, LM, BM) [2].

In this context, the “obesity paradox” has gained attention: while obesity is associated with higher bone mineral density (BMD) in some studies, it may also increase the risk of fractures [3, 4]. This paradox may stem from the opposing effects of mechanical loading and estrogen production from fat tissue, which can enhance BMD. In contrast, excess adiposity promotes inflammation and shifts mesenchymal stem cell differentiation toward adipogenesis over osteoblastogenesis, thereby impairing bone quality [5].

In children, although obesity may increase absolute BMD, it does not necessarily protect against fractures. After adjusting for body size, obese children often show compromised bone architecture, contributing to greater fracture risk, especially in the lower limbs [6].

Furthermore, not all fat has the same impact on bone [6, 7]. Visceral fat, in particular, is linked to adverse bone outcomes due to its inflammatory profile and hormonal disruptions [8].

A recent study in Chinese children found an inverse association between fat mass and BMD after adjusting for body weight [9], and a meta-analysis of 31 studies showed that LM has a stronger, more consistent positive association with BMD than FM, particularly in Asian populations and among boys [10].

Given the increasing prevalence of childhood obesity and its potential effect on skeletal health, it is essential to understand the relationship between FM, LM, and BMD. While previous research has highlighted the impact of body composition on bone health, there is still much to learn about the specific roles of fat distribution and LM in bone development during childhood and adolescence. This study aims to analyze the relationship between FM, LM, and central adiposity with BMD in healthy Mexican children and adolescents. We hypothesize that higher FM, increased waist circumference (WC), and lower LM will be associated with lower BMD, and that these effects will vary depending on fat distribution.

## Methods

### Study design and participants

We conducted a cross-sectional analysis based on the Health Workers Cohort Study, an ongoing prospective cohort study established in January 2004 to examine the association between lifestyle and genetic factors and chronic disease [11]. The HWCS includes health workers and their family members from three health institutions in Mexico: the Mexican Social Security Institute (IMSS), the National Institute of Public Health (INSP), and the Autonomous University of Mexico State (UAEM). These institutions, located in Cuernavaca and Toluca, Mexico, invited individuals aged 7–94 years to participate. Details of the study design and methods have been published previously [11]. For the present analysis, we included participants aged 7 to 21 years with available data on body composition and BMD, resulting in a final sample of 1,191 individuals.

We excluded the following participants from the analysis: incomplete dietary records (n = 42), implausible total energy intake values (i.e., < 600 or > 6000 kcal/day, n = 49 [12]), and individuals with missing Tanner stage information (n = 46). The final analytical sample consisted of 1,054 children and adolescents.

### Body composition assessment

Total LM, total FM, and truncal FM (TFM) were measured using dual-energy X-ray absorptiometry (DXA) (Lunar DPX NT), following established protocols and using a calibrated device. WC was assessed at the highest point of the iliac crest, using a flexible tape measure to the nearest 0.1 cm. BMD was evaluated using the DXA technique, with measurements taken in five anatomical regions: a) subtotal (including spine, ribs, pelvis, and extremities, but excluding the head), b) lumbar spine, c) hip, d) arm, and e) leg. The daily coefficient of variation was within standard operational limits, and the in vivo coefficient of variation was less than 1.0–1.5% [13].

### Covariates

Body weight was recorded using a calibrated electronic scale (model BC-533; Tanita), with participants wearing minimal clothing and no shoes. Height was measured using a standard stadiometer (Seca), with participants standing barefoot and in a neutral position. All anthropometric measurements were taken following standardized procedures [11].

Covariates, including age, sex, lifestyle factors (diet, physical activity, smoking status), and pubertal stage, were collected via self-administered questionnaires at baseline. For younger participants, parents or guardians completed the forms on their behalf. Age was grouped into three categories (< 10, 10–19, > 19 years) and also analyzed in tertiles when appropriate. Physical activity (PA) during leisure time was assessed using a validated 16-item questionnaire covering everyday activities (e.g., walking, running, cycling) [14]. Total leisure-time PA was calculated as the sum of frequency and duration of each activity divided by seven. PA was further classified according to WHO guidelines (≥ 1 h/day).

Dietary intake was assessed using a validated 116-item semi-quantitative food frequency questionnaire [15]. Nutrient intake and total energy (kcal/day) were estimated using food composition tables from the INSP [11]. The Dietary Inflammatory Index (DII), ranging from − 4.01 to 5.51, was calculated based on 30 food parameters derived from the FFQ using established methods [16], with higher scores indicating a more pro-inflammatory diet. Participants were classified according to smoking status as never, former, and current smokers. Sexual maturation in participants was assessed using the Tanner stages scale [17] through self-administered questionnaires that included images of secondary sexual characteristics. Participants selected the image that best represented their development. Those in stages I–IV were classified as pubertal, and those in stage V as post-pubertal [18].

### Statistical analyses

Descriptive statistics were used to summarize the characteristics of the study population. Means and standard deviations (SD) were calculated for continuous variables, while frequencies and percentages were reported for categorical variables. The data were stratified by sex (females and males) and pubertal status (pre-pubertal and post-pubertal).

To eliminate the effect of body weight on the exposures (FM, LM, TFM, and WC), independent linear regression models were first conducted for each exposure, using body weight (kg) as the independent variable [9, 19]. The residuals from these models represent the portion of each exposure that is independent of body weight. The weight-adjusted exposure values were obtained by adding the mean value of each exposure to its respective residual. This method enabled us to isolate the variation in each exposure that is not accounted for by body weight. The residual adjustment was performed separately for age groups (< 10, 10–19, and > 19 years) and sex. Following this, all exposure variables were standardized (using z-scores) to facilitate comparison of effect sizes across models and anatomical regions. These standardized, weight-adjusted values were subsequently used in all regression analyses.

A Directed Acyclic Graph was constructed a priori based on current biological knowledge and epidemiological evidence to guide the covariate selection and ensure a robust understanding of the assumed causal relationships between variables (Supplementary Fig. 1).

Separate multivariate linear regression models were fitted for each exposure variable to examine their associations with BMD. To avoid collinearity, exposure variables were not included in the same model simultaneously. Multicollinearity was explored using the Variance Inflation Factor (VIF) to assess potential issues with the inclusion of highly correlated predictor variables. Models were stratified by sex, age group, Tanner stage, DII categories and BMI categories, and adjusted for relevant potential confounders, including age (years), tanner stage, body weight (kg), physical activity during leisure time (inactive, active), dietary inflammatory index (tertile categories), calcium intake (EAR < 1000 mg, ≥ 1000 mg), and smoking status (never, past, current). Body weight was included in the regression models to control for its direct effect on BMD and any residual confounding not fully accounted for by the residual adjustment method, enhancing the validity of the estimates [19]. To assess heterogeneity by Tanner stage, DII categories, smoking status, weight categories defined by the median, physical activity, and calcium intake, interaction terms between these variables and the exposure variables were included in the models. All analyses were conducted using the final sample of 1,054 participants with complete data on exposures, outcomes, physical activity, and diet. For tobacco exposure, 42 participants had missing data; to retain them in the analysis, a separate "missing" category was included in the regression models. Additionally, a sensitivity analysis was conducted to explore the associations between the exposures and different BMD sites across tertiles. In this analysis, LM and FM were combined to examine their joint effects, forming three body composition groups based on tertiles for both males and females: Group 1 (high LM and low FM), Group 2 (similar LM and FM), and Group 3 (low LM and high FM). Linear regression models estimated average BMD values by sex across these groups. All statistical analyses were performed with the Stata program version 18.0 (Stata Corp, College Station, TX, USA), and significance was determined at a P-value < 0.05.

## Results

### Descriptive characteristics of the study population

The characteristics of our study population are shown in Table 1. The sample comprised 1,054 participants, with 437 males (372 pubertal and 65 post-pubertal) and 617 females (387 pubertal and 230 post-pubertal). The mean age of the participants was 14.7 years (SD = 4.1), with females comprising 58.8% of the sample. The characteristics of the participants varied by sex and pubertal status. Pubertal males and females were younger, lighter, and shorter than their post-pubertal counterparts.

Post-pubertal males and females showed higher fat and LM, with post-pubertal males having more fat and LM than pubertal males, and post-pubertal females showing significantly greater FMs and LM compared to pubertal females. Physical activity was low across all groups.

Calcium intake was suboptimal, with a significant proportion of both males and females failing to meet the recommended daily intake, particularly among post-pubertal individuals. The DII was slightly negative for both sexes, with pubertal males showing the least negative value. Tobacco use was significantly higher among post-pubertal participants, with more males and females reporting current smoking compared to their pubertal counterparts. A smaller percentage of pubertal participants were past smokers. BMD was significantly higher in post-pubertal individuals of both sexes. Post-pubertal males and females had notably higher total and site-specific BMD (lumbar spine, total hip, and leg) compared to pubertal individuals (Table 1).

### Association between body composition, WC, and BMD by sex

LM was positively associated with BMD across all anatomical regions in both males and females. In females, a one standard deviation increase in lean mass was associated with a 0.039 g/cm^2^ increase in hip BMD. The strongest associations were observed for leg and hip BMD. Conversely, FM showed consistent negative associations with BMD, particularly in total, subtotal, lumbar spine, and hip regions. TFM showed inconsistent associations overall, particularly among females. WC was inversely associated with BMD across nearly all skeletal regions of both sexes, with stronger effects observed in males (Table 2).

### Association between body composition, WC, and BMD by sex and pubertal status

Stratified analysis by pubertal status showed that LM maintained a positive association with BMD across all anatomical regions, with stronger associations during the pubertal stage compared to the post-pubertal stage. FM remained negatively associated with BMD, particularly during puberty, while associations in the post-pubertal stage were generally weaker. TFM had limited associations with BMD and appeared to exert more influence during puberty. WC was negatively associated with BMD across all regions, with stronger associations observed during puberty, especially in males (Table 3 and 4).

### Association between body composition, WC, and BMD by sex and age groups

When stratified by age groups and sex, LM was positively and consistently associated with BMD in adolescents. FM and WC also exhibited negative associations with BMD in both sexes, with more consistent and statistically significant associations observed in adolescents across several anatomical regions (Supplementary Tables 1 and 2). These trends persisted across age categories defined by tertiles, particularly in younger participants (Supplementary Tables 3 and 4).

### Association between body composition, WC, and BMD by BMI status

Stratified analyses by BMI status showed consistent positive associations between lean mass and BMD across weight categories, while fat mass and waist circumference remained inversely associated, with slightly weaker effects in the overweight/obese groups (Supplementary Tables 5 and 6).

### Association between body composition, WC, and BMD: Stratified by Lifestyle Factors

In males, significant interactions between DII categories and body composition were observed only for lumbar spine BMD. In females, truncal fat demonstrated a consistent inverse association with BMD, predominantly in the lowest DII tertile, except for arm BMD (Supplementary Tables 7 and 8). For other variables, such as physical activity, weight status, calcium intake, and smoking status, most interactions were not statistically significant, despite stronger effects observed in specific subgroups (Supplementary Tables 9 and 13).

### Multicollinearity evaluation

The VIF values did not exceed the threshold of 5, indicating that multicollinearity was not a significant issue in our models (Supplementary Table 14).

### Sensitivity analysis

**Fig. 1:**
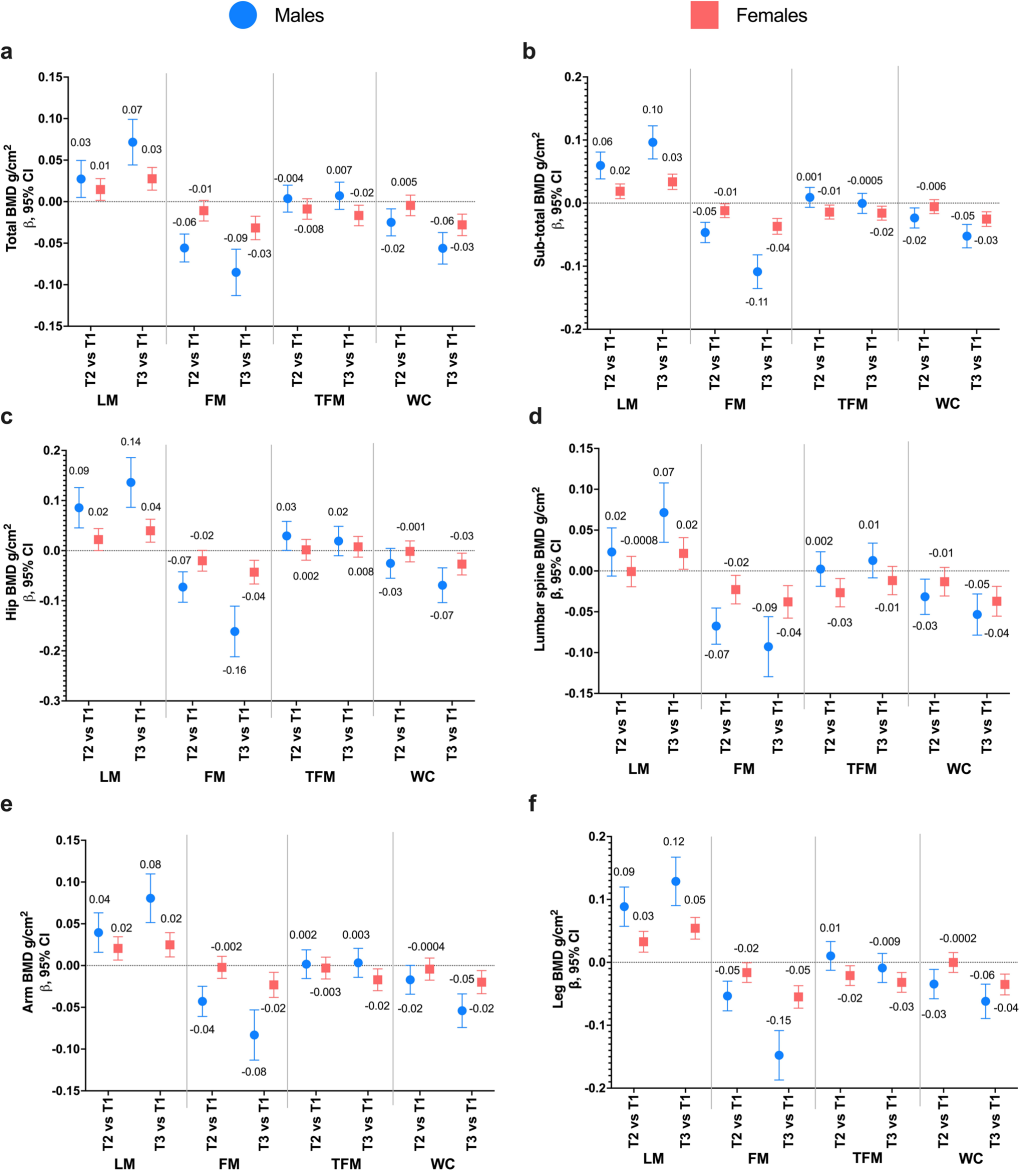
Association between Different Sites of Bone Mineral Density and Body Composition: Analysis by categories defined by tertiles of lean mass (LM), fat mass (FM), truncal fat mass (TFM), and waist circumference (WC). **a** Total BMD, **b** Sub-total BMD (excluding head), **c** Hip BMD, **d** Lumbar Spine BMD, **e** Arm BMD, and **f** Leg BMD. Models adjusted by age (years), dietary inflammatory index (continuous), physical activity leisure time (hours/week), calcium intake (EAR < 1000 mg, > 1000 mg), smoking status (never, past, and current), Tanner stage (five categorical stages: I to V), and weight(kg). To facilitate the interpretation of the results, we have used the labels T1, T2, and T3 for the categories defined by tertiles. However, it is important to note that these labels are convenient for presentation purposes, as the values of the tertiles define the low, medium, and high categories, respectively. Therefore, while we use this terminology, the tertiles represent value ranges

A sensitivity analysis that categorized exposures by tertiles yielded similar findings. Individuals in the highest tertile of LM had a higher average BMD across different anatomical sites. In contrast, those in the highest tertiles of FM and WC had lower average BMD compared to individuals in the lower tertiles. Notably, these associations were stronger in males (Fig. 1).

**Fig. 2:**
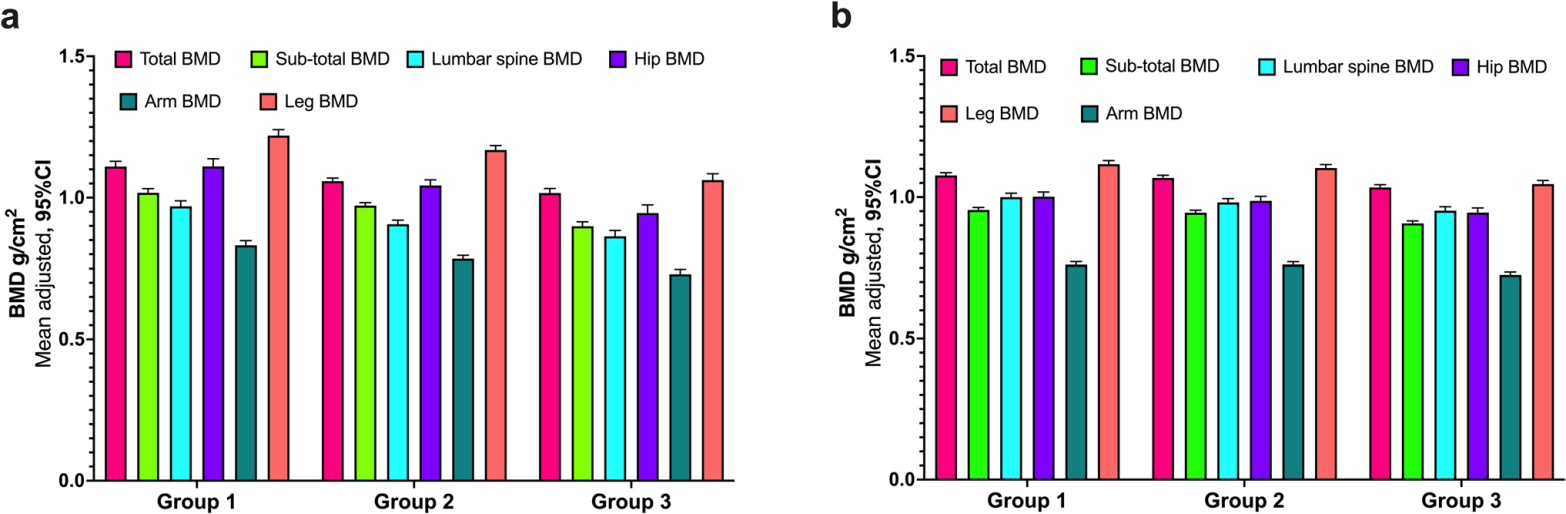
Average bone mineral density (BMD) at different anatomical sites according to different combinations of lean mass and fat mass. **a** Males, **b** Females. Models adjusted by age (years), dietary inflammatory index (continuous), physical activity leisure time (hours/week), calcium intake (EAR < 1000 mg, ≥ 1000 mg), smoking status (never, past, and current), Tanner stage (five categorical stages: I to V), and weight(kg). The three body composition groups were created based on tertiles of lean mass (LM), and fat mass (FM) for boys and girls. Group 1 consisted of participants with high lean mass and low-fat mass values (*n* = 322). Group 2 included participants with similar values of lean mass and fat mass (*n* = 311). Group 3 comprised participants with low lean mass and high fat mass values (*n* = 326). The data presented are from linear regression models using margins at means to estimate average bone mineral density (BMD) at different anatomical sites. The exposure variables were not standardized

Additionally, we observed that both males and females in Group 1 (those with higher LM and lower FM) had, on average, higher BMD at all anatomical sites compared to Group 3 (those with lower LM and higher FM) (Fig. 2).

## Discussion

Our findings highlight the significant role of LM in promoting BMD, particularly in individuals going through puberty, with notable variations observed across sex, pubertal stage, and age. Conversely, FM was consistently found to be inversely associated with BMD. Sensitivity analyses confirmed that participants with higher LM and lower FM had the highest BMD.

Our findings are consistent with previous studies reporting the positive impact of LM and the negative influence of FM on bone health in pediatric populations [10, 20, 21]. Similar to findings in Chinese children [9], FM was inversely associated with BMD after adjusting for weight using a residuals-based method, which we employed to reduce collinearity and isolate FM effects more precisely [22]. Stronger associations in males likely reflect higher LM, greater mechanical loading, and sex-specific hormonal profiles [10, 21].

LM likely supports bone health through mechanical loading and endocrine factors, particularly insulin-like growth factor 1 (IGF-1), which promotes osteoblast activity and bone mineralization [23, 24].

Conversely, excess FM may impair bone by promoting inflammation and increasing osteoclast-mediated resorption, particularly in cases of abdominal adiposity [25–27]. Hormonal changes during puberty, especially in girls, may further modulate these effects [27–29]. Pubertal changes in fat distribution and fluctuations in estrogen, FSH, and androgens may modulate the impact of FM on bone. Additionally, abdominal adiposity has been linked to elevated secretion of visceral fat–derived cytokines, further contributing to bone loss [28, 30–32].

Taken together, these results emphasize the importance of maintaining adequate LM while minimizing excessive FM during critical periods of bone development. Muscle mass appears to have protective effects on bone, both biomechanically and through endocrine pathways, whereas FM, particularly in central regions, may negatively impact bone integrity.

## Strengths

This study has several strengths. First, the large and diverse sample, encompassing various developmental stages and both sexes, allowed for robust subgroup analyses. Second, BMD was assessed using dual-energy X-ray absorptiometry (DXA), the gold standard, across multiple anatomical regions, enabling a detailed understanding of regional bone effects. Third, the use of a residual-based adjustment for body weight minimized collinearity and improved the estimation of the independent contributions of LM and FM. Finally, this study provides timely insights into the context of the obesity epidemic and its implications for bone and metabolic health.

## Limitations

However, some limitations should be acknowledged. Due to the cross-sectional design, causality and temporal relationships between body composition and BMD cannot be established. Although we adjusted for key confounders, such as age, physical activity, calcium intake, and pubertal stage, residual confounding remains possible. For instance, our assessment of physical activity was limited to leisure time and did not include school-based or organized sports, which may underestimate total physical activity. Additionally, participants in the Health Workers Cohort Study may not be representative of the broader pediatric population, which limits the generalizability of the findings to other demographic or socioeconomic groups.

The findings of this study highlight the importance of maintaining an optimal body composition during childhood and adolescence, particularly by increasing LM through physical activity to support bone health. Additionally, puberty is a critical period for bone development, making it essential to implement interventions that promote healthy body composition and minimize excess fat. Such measures could provide long-term benefits for bone health and help prevent fractures.

## Conclusion

In conclusion, this study provides valuable insights into the relationship between body composition and bone health in growing children and adolescents. Future research should investigate the underlying mechanisms that link body composition to bone mineralization, with a particular focus on the roles of hormones, growth factors, and mechanical loading. Longitudinal studies that assess the long-term effects of body composition on bone health during childhood and adolescence would be beneficial for understanding the trajectory of bone development and identifying potential risk factors for osteoporosis later in life.

## Supplementary information

Below are the links to the electronic supplementary materials.

Supplementary file 1 (JPEG 123 KB)

Supplementary file 2 (DOCX 112 KB)

## Data Availability

The datasets used and analyzed during the study are available from the corresponding author on reasonable request.

## List of abbreviations

BMI: Body mass index;
BMD: Bone mineral density;
BMC: Bone mineral content;
DXA: Dual-energy X-ray absorptiometry;
DII: Dietary Inflammation Index;
IGF-1: Insulin-like growth factor-1;
FM: Fat mass;
LM: Lean mass;
LT: Leisure time;
PA: Physical activity;
TNF-α: Tumor necrosis factor-α;
TFM: Truncal Fat Mass;
WC: Waist circumference
