# Supplementary figures and images for: Association between lean mass, fat mass, and waist circumference with bone mineral density in Mexican children and adolescents: a cross-sectional study
Source: Eur J Pediatr. 2025 Oct 9;184(11):671. doi: 10.1007/s00431-025-06515-9 (PMC12511192; doi:10.1007/s00431-025-06515-9)

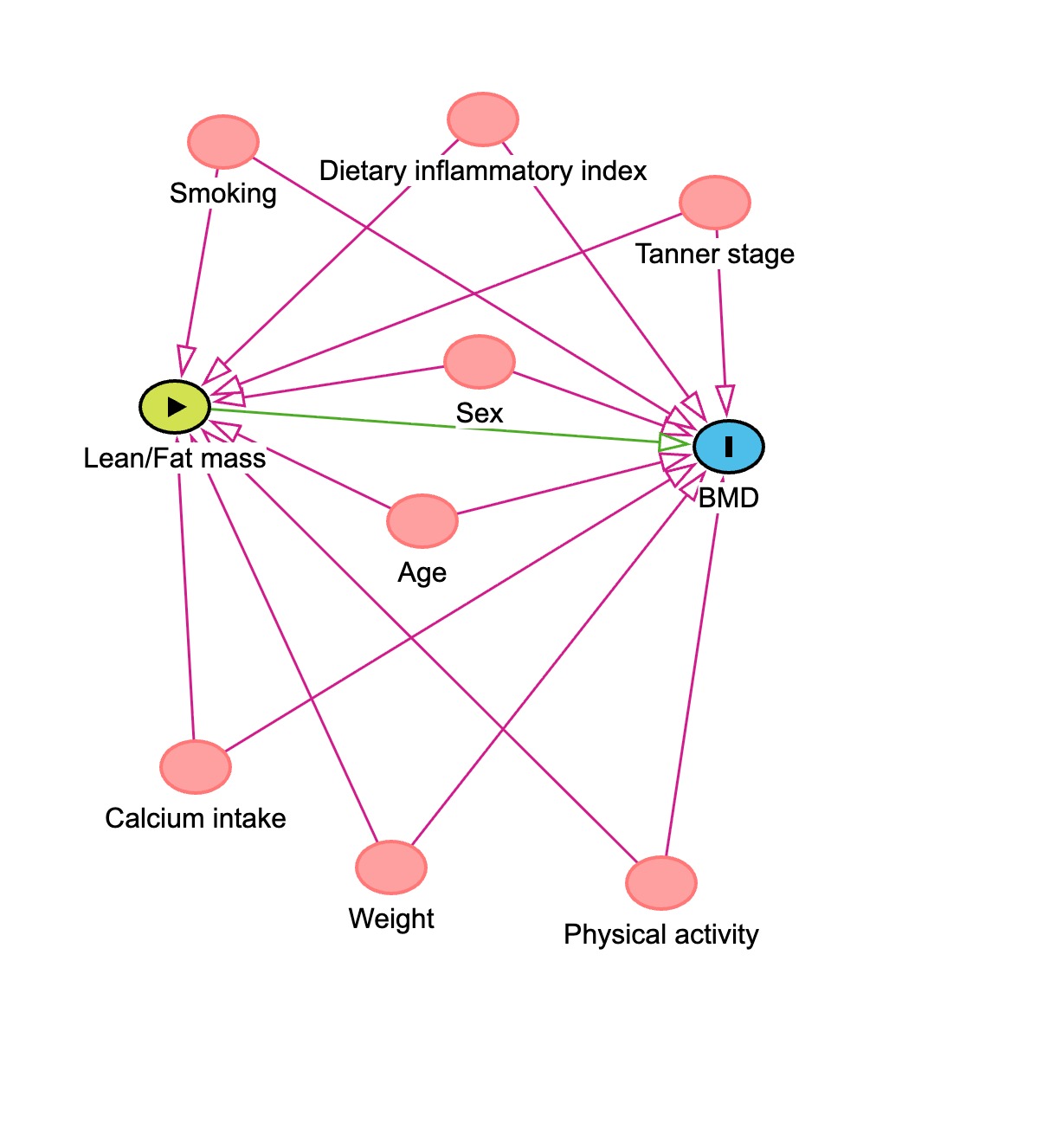

Supplement: Supplementary file 1 — (JPEG 123 KB) [file 431_2025_6515_MOESM1_ESM.jpeg]
