# Supplementary material for: Association between lean mass, fat mass, and waist circumference with bone mineral density in Mexican children and adolescents: a cross-sectional study
Source: Eur J Pediatr. 2025 Oct 9;184(11):671. doi: 10.1007/s00431-025-06515-9 (PMC12511192; doi:10.1007/s00431-025-06515-9)
Supplement: Supplementary file 2 — (DOCX 112 KB) [file 431_2025_6515_MOESM2_ESM.docx]

**Association between lean mass, fat mass, and waist circumference with bone mineral density in Mexican children and adolescents**

Berenice Rivera-Paredez ^1α*^, Karla Muciño-Sandoval ^2α^, Rafael Velázquez-Cruz^3^, Rodolfo Rivas-Ruíz^4^, Joacim Meneses-León^1,5^, Ricardo Orozco^6^, Carlos Esteban González Muñoz^1^, Juan Tamayo-Orozco^1^, Edgar Denova-Gutiérrez ^7^, Jorge Salmerón^1^, Marcela Tamayo-Ortiz^8*^

^α^ Contributed equally.

^1^ Research Center on Policies, Population and Health, Faculty of Medicine, National Autonomous University of Mexico, Mexico City, Mexico.

^2^ Accessalud, Mexico City, Mexico.

^3^ Laboratory of Genomics of Bone Metabolism, National Institute of Genomic Medicine. Mexico City, Mexico.

^4^ Training and Clinical Research Center. Health Research Coordination. Mexican Social Security Institute. Mexico City, Mexico.

^5^ Center for Evaluation and Survey Research, National Institute for Public Health, Cuernavaca, Morelos, Mexico.

^6^ Global Mental Health Research Center, Ramón de la Fuente National Institute of Psychiatry, Mexico City, Mexico.

^7^ Nutrition Department, National Institute of Medical Sciences and Nutrition Salvador Zubirán, Mexico City, Mexico.

^8^ Department of Environmental Health Sciences, Mailman School of Public Health at Columbia University, New York, United States.

^*^**Corresponding authors:**

Berenice Rivera-Paredez

Research Center on Policies, Population and Health, Faculty of Medicine, National Autonomous University of Mexico, Ciudad Universitaria, Edificio CIPPS-Sótano y Piso 2, Cto. Centro Cultural S/N, Coyoacán, 04510 Mexico City, Mexico

Email: [bereriveraparedez7@gmail.com](mailto:bereriveraparedez7@gmail.com)

Marcela Tamayo-Ortiz

Department of Environmental Health Sciences, Mailman School of Public Health at Columbia University, 722 W 168th St, New York, NY 10032, United States.

Email: mt3743@cumc.columbia.edu

| **Supplementary Table 1**. Associations between lean mass (kg), fat mass (kg) and bone mineral density (g/cm^2^) in different anatomical regions, by age group in **male** of the Health Workers Cohort Study. | | | |
| --- | --- | --- | --- |
|  | **Children**  **(n= 60)** | **Adolescents**  **(n= 326)** | **Young adults**  **(n= 51)** |
|  | β (95% CI) | β (95% CI) | β (95% CI) |
| **Total BMD,** g/cm^2^ |  |  |  |
| Lean mass | 0.013 (-0.048,0.074) | 0.048 (0.037,0.060) | 0.042 (-0.001,0.085) |
| Fat mass | -0.008 (-0.053,0.038) | -0.053 (-0.064,-0.041) | -0.039 (-0.078,0.001) |
| Truncal fat mass | 0.001 (-0.016,0.019) | -0.008 (-0.015,-0.0004) | -0.002 (-0.026,0.022) |
| Waist Circumference | 0.017 (-0.005,0.039) | -0.033 (-0.042,-0.023) | -0.003 (-0.032,0.026) |
| **Sub-total BMD,** g/cm^2^ |  |  |  |
| Lean mass | 0.035 (-0.023,0.092) | 0.058 (0.048,0.069) | 0.045 (0.001,0.090) |
| Fat mass | -0.041 (-0.083,0.0005) | -0.062 (-0.074,-0.051) | -0.042 (-0.083,-0.002) |
| Truncal fat mass | 0.003 (-0.013,0.020) | -0.009 (-0.016,-0.001) | -0.005 (-0.029,0.020) |
| Waist Circumference | -0.002 (-0.023,0.020) | -0.033 (-0.042,-0.024) | -0.002 (-0.032,0.027) |
| **Lumbar Spine BMD,** g/cm^2^ |  |  |  |
| Lean mass | 0.037 (-0.027,0.100) | 0.053 (0.039,0.068) | 0.068 (0.001,0.134) |
| Fat mass | -0.042 (-0.088,0.004) | -0.059 (-0.074,-0.044) | -0.090 (-0.147,-0.033) |
| Truncal fat mass | 0.012 (-0.006,0.030) | -0.012 (-0.021,-0.003) | -0.029 (-0.065,0.006) |
| Waist Circumference | -0.008 (-0.031,0.016) | -0.032 (-0.043,-0.020) | -0.005 (-0.050,0.041) |
| **Hip BMD,** g/cm^2^ |  |  |  |
| Lean mass | 0.045 (-0.082,0.172) | 0.082 (0.061,0.103) | 0.058 (-0.029,0.146) |
| Fat mass | -0.081 (-0.173,0.011) | -0.088 (-0.110,-0.067) | -0.056 (-0.136,0.023) |
| Truncal fat mass | -0.006 (-0.042,0.031) | -0.007 (-0.021,0.006) | 0.004 (-0.043,0.052) |
| Waist Circumference | -0.005 (-0.052,0.042) | -0.044 (-0.061,-0.027) | 0.016 (-0.041,0.073) |
| **Arm BMD,** g/cm^2^ |  |  |  |
| Lean mass | -0.010 (-0.065,0.044) | 0.051 (0.039,0.063) | 0.044 (-0.002,0.091) |
| Fat mass | 0.011 (-0.029,0.052) | -0.052 (-0.065,-0.039) | -0.035 (-0.078,0.008) |
| Truncal fat mass | -0.005 (-0.021,0.010) | -0.009 (-0.017,-0.001) | -0.005 (-0.030,0.021) |
| Waist Circumference | 0.014 (-0.006,0.034) | -0.032 (-0.042,-0.022) | -0.020 (-0.051,0.011) |
| **Leg BMD,** g/cm^2^ |  |  |  |
| Lean mass | 0.040 (-0.043,0.122) | 0.077 (0.060,0.094) | 0.054 (-0.007,0.115) |
| Fat mass | -0.060 (-0.119,-0.0004) | -0.084 (-0.101,-0.067) | -0.052 (-0.108,0.003) |
| Truncal fat mass | -0.0001 (-0.024,0.024) | -0.013 (-0.024,-0.002) | -0.004 (-0.037,0.030) |
| Waist Circumference | 0.002 (-0.028,0.033) | -0.042 (-0.056,-0.028) | 0.002 (-0.038,0.042) |
| Models adjusted by age (years), dietary inflammatory index (categories defined by tertiles), physical activity leisure time (hours/week), calcium intake (EAR <1000, >1000 mg), smoking status (never, past and actual), tanner stage (five categorical stages: I to V) and weight(kg). Children <10 years, Adolescents 10-19 years, Young adults 20-21 years. Exposure variables were standardized (z-scores) | | | |

| **Supplementary Table 2**. Associations between lean mass (kg), fat mass (kg) and bone mineral density (g/cm^2^) in different anatomical regions, by age group in **female** of the Health Workers Cohort Study. | | | |
| --- | --- | --- | --- |
|  | **Children**  **(n= 70)** | **Adolescents**  **(n= 429)** | **Young adults**  **(n= 118)** |
|  | β (95% CI) | β (95% CI) | β (95% CI) |
| **Total BMD,** g/cm^2^ |  |  |  |
| Lean mass | 0.021 (-0.023,0.064) | 0.035 (0.019,0.052) | -0.0005 (-0.032,0.031) |
| Fat mass | -0.023 (-0.065,0.019) | -0.042 (-0.059,-0.026) | -0.005 (-0.036,0.025) |
| Truncal fat mass | 0.003 (-0.004,0.011) | -0.009 (-0.016,-0.001) | 0.001 (-0.011,0.012) |
| Waist Circumference | -0.002 (-0.018,0.014) | -0.018 (-0.025,-0.010) | -0.009 (-0.021,0.003) |
| **Sub-total BMD,** g/cm^2^ |  |  |  |
| Lean mass |  |  |  |
| Fat mass | -0.031 (-0.077,0.015) | -0.047 (-0.061,-0.033) | -0.013 (-0.041,0.015) |
| Truncal fat mass | 0.016 (0.008,0.023) | -0.010 (-0.016,-0.004) | 0.002 (-0.009,0.012) |
| Waist Circumference | 0.001 (-0.017,0.019) | -0.017 (-0.024,-0.011) | -0.010 (-0.020,0.001) |
| **Lumbar Spine BMD,** g/cm^2^ |  |  |  |
| Lean mass | 0.015 (-0.056,0.086) | 0.019 (-0.004,0.042) | -0.009 (-0.061,0.043) |
| Fat mass | 0.001 (-0.068,0.070) | -0.043 (-0.065,-0.020) | -0.037 (-0.087,0.013) |
| Truncal fat mass | 0.034 (0.025,0.043) | -0.017 (-0.027,-0.007) | -0.016 (-0.035,0.002) |
| Waist Circumference | 0.008 (-0.018,0.034) | -0.018 (-0.029,-0.008) | -0.031 (-0.050,-0.013) |
| **Hip BMD,** g/cm^2^ |  |  |  |
| Lean mass | 0.030 (-0.048,0.108) | 0.049 (0.022,0.077) | 0.007 (-0.045,0.059) |
| Fat mass | -0.022 (-0.098,0.053) | -0.061 (-0.088,-0.033) | -0.009 (-0.059,0.041) |
| Truncal fat mass | 0.007 (-0.007,0.021) | 0.002 (-0.010,0.013) | 0.019 (0.001,0.038) |
| Waist Circumference | -0.006 (-0.035,0.022) | -0.017 (-0.030,-0.004) | -0.006 (-0.026,0.013) |
| **Arm BMD,** g/cm^2^ |  |  |  |
| Lean mass | 0.016 (-0.106,0.139) | 0.024 (0.010,0.039) | 0.027 (-0.008,0.062) |
| Fat mass | -0.044 (-0.161,0.073) | -0.030 (-0.044,-0.015) | -0.030 (-0.063,0.004) |
| Truncal fat mass | -0.071 (-0.082,-0.059) | -0.007 (-0.013,-0.0004) | -0.002 (-0.015,0.011) |
| Waist Circumference | -0.016 (-0.060,0.029) | -0.015 (-0.021,-0.008) | -0.006 (-0.019,0.007) |
| **Leg BMD,** g/cm^2^ |  |  |  |
| Lean mass | 0.086 (0.026,0.145) | 0.063 (0.042,0.083) | 0.034 (-0.005,0.072) |
| Fat mass | -0.052 (-0.112,0.008) | -0.061 (-0.082,-0.041) | -0.035 (-0.072,0.002) |
| Truncal fat mass | -0.007 (-0.018,0.004) | -0.008 (-0.017,0.001) | 0.001 (-0.014,0.015) |
| Waist Circumference | -0.005 (-0.028,0.019) | -0.025 (-0.034,-0.015) | -0.014 (-0.028,0.00004) |
| Models adjusted by age (years), dietary inflammatory index (categories defined by tertiles), physical activity leisure time (hours/week), calcium intake (EAR <1000 mg, >1000 mg), smoking status (never, past and actual), tanner stage (five categorical stages: I to V) and weight(kg). Children <10 years, Adolescents 10-19 years, Young adults 20-21 years. Exposure variables were standardized (z-scores) | | | |

| **Supplementary Table 3**. Associations between lean mass (kg), fat mass (kg) and bone mineral density (g/cm^2^) in different anatomical regions, by age group in **male** of the Health Workers Cohort Study. | | | |
| --- | --- | --- | --- |
|  | **Age categories defined by tertiles** | | |
|  | **Category low**  **7-12 years**  **(n= 160)** | **Category medium**  **13-16 years**  **(n= 140)** | **Category high**  **17-21 years**  **(n= 137)** |
|  | β (95% CI) | β (95% CI) | β (95% CI) |
| **Total BMD,** g/cm^2^ |  |  |  |
| Lean mass | 0.002 (-0.024,0.027) | 0.016 (-0.007,0.040) | 0.038 (0.015,0.061) |
| Fat mass | -0.037 (-0.058,-0.016) | -0.062 (-0.079,-0.044) | -0.033 (-0.057,-0.010) |
| Truncal fat mass | -0.00007 (-0.013,0.013) | -0.016 (-0.035,0.002) | -0.003 (-0.011,0.006) |
| Waist Circumference | -0.012 (-0.025,0.0001) | -0.047 (-0.061,-0.033) | -0.009 (-0.027,0.010) |
| **Sub-total BMD,** g/cm^2^ |  |  |  |
| Lean mass | 0.057 (0.039,0.076) | 0.065 (0.049,0.082) | 0.029 (0.006,0.053) |
| Fat mass | -0.060 (-0.079,-0.042) | -0.069 (-0.086,-0.053) | -0.032 (-0.055,-0.009) |
| Truncal fat mass | 0.001 (-0.012,0.013) | -0.016 (-0.035,0.003) | -0.004 (-0.013,0.005) |
| Waist Circumference | -0.021 (-0.032,-0.010) | -0.048 (-0.062,-0.034) | -0.005 (-0.023,0.013) |
| **Lumbar Spine BMD,** g/cm^2^ |  |  |  |
| Lean mass | 0.034 (0.011,0.056) | 0.065 (0.044,0.085) | 0.033 (-0.004,0.069) |
| Fat mass | -0.040 (-0.062,-0.018) | -0.070 (-0.090,-0.049) | -0.052 (-0.088,-0.017) |
| Truncal fat mass | 0.013 (-0.001,0.027) | -0.003 (-0.025,0.020) | -0.018 (-0.031,-0.005) |
| Waist Circumference | -0.017 (-0.029,-0.004) | -0.053 (-0.069,-0.036) | -0.006 (-0.034,0.022) |
| **Hip BMD,** g/cm^2^ |  |  |  |
| Lean mass | 0.078 (0.039,0.117) | 0.094 (0.061,0.126) | 0.040 (-0.003,0.084) |
| Fat mass | -0.083 (-0.122,-0.045) | -0.101 (-0.134,-0.069) | -0.046 (-0.089,-0.003) |
| Truncal fat mass | 0.006 (-0.018,0.031) | -0.012 (-0.047,0.022) | -0.002 (-0.018,0.014) |
| Waist Circumference | -0.032 (-0.055,-0.010) | -0.067 (-0.093,-0.040) | 0.009 (-0.025,0.043) |
| **Arm BMD,** g/cm^2^ |  |  |  |
| Lean mass | 0.041 (0.022,0.061) | 0.064 (0.045,0.083) | 0.028 (0.002,0.055) |
| Fat mass | -0.037 (-0.057,-0.018) | -0.066 (-0.085,-0.047) | -0.026 (-0.052,0.001) |
| Truncal fat mass | -0.002 (-0.015,0.010) | -0.019 (-0.039,0.002) | -0.005 (-0.015,0.005) |
| Waist Circumference | -0.014 (-0.025,-0.003) | -0.048 (-0.064,-0.033) | -0.016 (-0.037,0.004) |
| **Leg BMD,** g/cm^2^ |  |  |  |
| Lean mass | 0.083 (0.054,0.111) | 0.082 (0.057,0.107) | 0.034 (0.0004,0.067) |
| Fat mass | -0.090 (-0.117,-0.062) | -0.088 (-0.113,-0.063) | -0.040 (-0.073,-0.007) |
| Truncal fat mass | -0.005 (-0.024,0.014) | -0.024 (-0.051,0.003) | -0.004 (-0.016,0.009) |
| Waist Circumference | -0.023 (-0.040,-0.006) | -0.060 (-0.081,-0.040) | -0.004 (-0.030,0.022) |
| Models adjusted by age (years), dietary inflammatory index (categories defined by tertiles), physical activity leisure time (hours/week), calcium intake (EAR <1000mg, >1000 mg), smoking status (never, past and actual), tanner stage (five categorical stages: I to V), and weight(kg). Children <10 years, Adolescents 10-19 years, Young adults 20-21 years. Exposure variables were standardized (z-scores) | | | |

| **Supplementary Table 4**. Associations between lean mass (kg), fat mass (kg) and bone mineral density (g/cm^2^) in different anatomical regions, by age group in **female** of the Health Workers Cohort Study. | | | |
| --- | --- | --- | --- |
|  | **Age categories defined by tertiles** | | |
|  | **Category low**  **7-13 years**  **(n= 225)** | **Category medium**  **14-18 years**  **(n= 220)** | **Category high**  **19-21 years**  **(n= 172)** |
|  | β (95% CI) | β (95% CI) | β (95% CI) |
| **Total BMD,** g/cm^2^ |  |  |  |
| Lean mass | 0.030 (0.006,0.054) | 0.016 (-0.007,0.040) | 0.002 (-0.024,0.027) |
| Fat mass | -0.043 (-0.067,-0.020) | -0.026 (-0.048,-0.003) | -0.010 (-0.035,0.016) |
| Truncal fat mass | -0.002 (-0.009,0.005) | -0.010 (-0.021,0.00007) | -0.003 (-0.013,0.008) |
| Waist Circumference | -0.022 (-0.032,-0.012) | -0.011 (-0.022,-0.001) | -0.010 (-0.019,0.0002) |
| **Sub-total BMD,** g/cm^2^ |  |  |  |
| Lean mass | 0.047 (0.027,0.067) | 0.023 (0.002,0.043) | 0.012 (-0.011,0.035) |
| Fat mass | -0.057 (-0.078,-0.037) | -0.028 (-0.047,-0.008) | -0.018 (-0.041,0.005) |
| Truncal fat mass | -0.0004 (-0.007,0.006) | -0.009 (-0.018,0.0004) | -0.001 (-0.011,0.008) |
| Waist Circumference | -0.016 (-0.026,-0.007) | -0.011 (-0.020,-0.002) | -0.011 (-0.019,-0.002) |
| **Lumbar Spine BMD,** g/cm^2^ |  |  |  |
| Lean mass | 0.013 (-0.019,0.045) | 0.005 (-0.027,0.036) | -0.019 (-0.060,0.021) |
| Fat mass | -0.060 (-0.092,-0.028) | -0.013 (-0.044,0.018) | -0.021 (-0.061,0.019) |
| Truncal fat mass | -0.003 (-0.012,0.007) | -0.011 (-0.025,0.003) | -0.011 (-0.027,0.006) |
| Waist Circumference | -0.022 (-0.036,-0.008) | -0.007 (-0.022,0.007) | -0.028 (-0.043,-0.013) |
| **Hip BMD,** g/cm^2^ |  |  |  |
| Lean mass | 0.065 (0.028,0.102) | 0.027 (-0.012,0.066) | 0.005 (-0.040,0.051) |
| Fat mass | -0.074 (-0.113,-0.036) | -0.036 (-0.074,0.001) | -0.013 (-0.058,0.031) |
| Truncal fat mass | -0.00001 (-0.012,0.012) | 0.007 (-0.010,0.024) | 0.017 (-0.001,0.035) |
| Waist Circumference | -0.021 (-0.038,-0.004) | -0.010 (-0.028,0.007) | -0.008 (-0.026,0.010) |
| **Arm BMD,** g/cm^2^ |  |  |  |
| Lean mass | 0.035 (0.005,0.064) | 0.008 (-0.012,0.028) | 0.020 (-0.007,0.048) |
| Fat mass | -0.050 (-0.080,-0.019) | -0.012 (-0.031,0.007) | -0.023 (-0.050,0.004) |
| Truncal fat mass | -0.035 (-0.043,-0.027) | 0.0002 (-0.009,0.009) | -0.008 (-0.019,0.003) |
| Waist Circumference | -0.014 (-0.027,-0.001) | -0.009 (-0.018,-0.001) | -0.006 (-0.016,0.005) |
| **Leg BMD,** g/cm^2^ |  |  |  |
| Lean mass | 0.084 (0.057,0.111) | 0.036 (0.006,0.065) | 0.038 (0.006,0.069) |
| Fat mass | -0.067 (-0.096,-0.038) | -0.041 (-0.069,-0.012) | -0.044 (-0.076,-0.013) |
| Truncal fat mass | -0.004 (-0.013,0.005) | -0.017 (-0.030,-0.004) | -0.003 (-0.016,0.010) |
| Waist Circumference | -0.017 (-0.031,-0.004) | -0.019 (-0.033,-0.006) | -0.016 (-0.028,-0.004) |
| Models adjusted by age (years), dietary inflammatory index (categories defined by tertiles), physical activity leisure time (hours/week), calcium intake (EAR <1000mg, >1000 mg), smoking status (never, past and actual), tanner stage (five categorical stages: I to V), and weight(kg). Children <10 years, Adolescents 10-19 years, Young adults 20-21 years. Exposure variables were standardized (z-scores) | | | |

| **Supplementary Table 5**. Associations between lean mass (kg), fat mass (kg) and bone mineral density (g/cm^2^) in different anatomical regions, by BMI categories in **male** of the Health Workers Cohort Study. | | |
| --- | --- | --- |
|  | **Normal weight**  **n=290** | **Overweight/obesity**  **n=147** |
|  | β (95% CI) | β (95% CI) |
| **Total BMD,** g/cm^2^ |  |  |
| Lean mass | 0.030 (0.014,0.046) | 0.059 (0.040,0.078) |
| Fat mass | -0.035 (-0.051,-0.019 | -0.063 (-0.083,-0.043) |
| Truncal fat mass | 0.002 (-0.010,0.015) | -0.005 (-0.014,0.004) |
| Waist Circumference | -0.019 (-0.031,-0.007) | -0.040 (-0.054,-0.026) |
| **Sub-total BMD,** g/cm^2^ |  |  |
| Lean mass | 0.040 (0.025,0.054) | 0.067 (0.048,0.086) |
| Fat mass | -0.044 (-0.058,-0.029) | -0.071 (-0.090,-0.052) |
| Truncal fat mass | -0.002 (-0.013,0.010) | -0.006 (-0.015,0.003) |
| Waist Circumference | -0.019 (-0.030,-0.008) | -0.040 (-0.054,-0.026) |
| **Lumbar Spine BMD,** g/cm^2^ |  |  |
| Lean mass | 0.027 (0.008,0.047) | 0.061 (0.033,0.089) |
| Fat mass | -0.038 (-0.058,-0.019) | -0.075 (-0.103,-0.048) |
| Truncal fat mass | 0.005 (-0.010,0.020) | -0.010 (-0.022,0.002) |
| Waist Circumference | -0.020 (-0.035,-0.006) | -0.043 (-0.062,-0.023) |
| **Hip BMD,** g/cm^2^ |  |  |
| Lean mass | 0.061 (0.032,0.090) | 0.100 (0.065,0.135) |
| Fat mass | -0.071 (-0.099,-0.042) | -0.106 (-0.141,-0.070) |
| Truncal fat mass | -0.001 (-0.023,0.022) | -0.002 (-0.018,0.014) |
| Waist Circumference | -0.030 (-0.052,-0.008) | -0.049 (-0.075,-0.023) |
| **Arm BMD,** g/cm^2^ |  |  |
| Lean mass | 0.029 (0.013,0.046) | 0.070 (0.049,0.092) |
| Fat mass | -0.027 (-0.044,-0.011) | -0.072 (-0.093,-0.050) |
| Truncal fat mass | 0.007 (-0.006,0.019) | -0.009 (-0.019,0.001) |
| Waist Circumference | -0.014 (-0.026,-0.002) | -0.051 (-0.066,-0.037) |
| **Leg BMD,** g/cm^2^ |  |  |
| Lean mass | 0.052 (0.031,0.074) | 0.085 (0.057,0.112) |
| Fat mass | -0.060 (-0.081,-0.038) | -0.090 (-0.118,-0.062) |
| Truncal fat mass | -0.009 (-0.026,0.008) | -0.007 (-0.020,0.005) |
| Waist Circumference | -0.024 (-0.040,-0.008) | -0.047 (-0.067,-0.027) |
| Models adjusted by age (years), dietary inflammatory index (categories defined by tertiles), physical activity leisure time (hours/week), calcium intake (EAR <1000mg, >1000 mg), weight (kg), smoking status (never, past and actual) and tanner stage (five categorical stages: I to V). Exposure variables were standardized (z-scores) | | |

| **Supplementary Table 6**. Associations between lean mass (kg), fat mass (kg) and bone mineral density (g/cm^2^) in different anatomical regions, by BMI categories in **female** of the Health Workers Cohort Study. | | |
| --- | --- | --- |
|  | **Normal weight**  **n=422** | **Overweight/obesity**  **n=195** |
|  | β (95% CI) | β (95% CI) |
| **Total BMD,** g/cm^2^ |  |  |
| Lean mass | 0.015 (-0.003,0.033) | 0.039 (0.015,0.064) |
| Fat mass | -0.022 (-0.040,-0.003) | -0.041 (-0.065,-0.017) |
| Truncal fat mass | 0.001 (-0.005,0.008) | -0.006 (-0.016,0.003) |
| Waist Circumference | -0.012 (-0.020,-0.005) | -0.014 (-0.025,-0.004) |
| **Sub-total BMD,** g/cm^2^ |  |  |
| Lean mass | 0.028 (0.012,0.043) | 0.042 (0.020,0.065) |
| Fat mass | -0.036 (-0.052,-0.020) | -0.041 (-0.063,-0.020) |
| Truncal fat mass | 0.003 (-0.003,0.009) | -0.007 (-0.016,0.001) |
| Waist Circumference | -0.010 (-0.017,-0.004) | -0.015 (-0.025,-0.004) |
| **Lumbar Spine BMD,** g/cm^2^ |  |  |
| Lean mass | -0.016 (-0.039,0.007) | 0.029 (-0.010,0.068) |
| Fat mass | -0.023 (-0.047,0.001) | -0.042 (-0.078,-0.005) |
| Truncal fat mass | -0.001 (-0.010,0.007) | -0.013 (-0.028,0.001) |
| Waist Circumference | -0.013 (-0.023,-0.004) | -0.034 (-0.050,-0.017) |
| **Hip BMD,** g/cm^2^ |  |  |
| Lean mass | 0.027 (-0.003,0.056) | 0.050 (0.008,0.092) |
| Fat mass | -0.042 (-0.072,-0.012) | -0.044 (-0.084,-0.003) |
| Truncal fat mass | 0.010 (-0.001,0.020) | 0.007 (-0.009,0.023) |
| Waist Circumference | -0.012 (-0.024,0.001) | -0.010 (-0.029,0.009) |
| **Arm BMD,** g/cm^2^ |  |  |
| Lean mass | 0.023 (0.003,0.043) | 0.036 (0.013,0.059) |
| Fat mass | -0.030 (-0.050,-0.009) | -0.035 (-0.057,-0.013) |
| Truncal fat mass | -0.022 (-0.029,-0.015) | -0.005 (-0.013,0.004) |
| Waist Circumference | -0.008 (-0.016,0.001) | -0.014 (-0.024,-0.004) |
| **Leg BMD,** g/cm^2^ |  |  |
| Lean mass | 0.055 (0.033,0.077) | 0.058 (0.027,0.090) |
| Fat mass | -0.051 (-0.074,-0.028) | -0.054 (-0.084,-0.024) |
| Truncal fat mass | 0.0002 (-0.008,0.008) | -0.011 (-0.023,0.001) |
| Waist Circumference | -0.014 (-0.023,-0.004) | -0.021 (-0.035,-0.007) |
| Models adjusted by age (years), dietary inflammatory index (categories defined by tertiles), physical activity leisure time (hours/week), calcium intake (EAR <1000mg, >1000 mg), weight (kg), smoking status (never, past and actual) and tanner stage (five categorical stages: I to V). Exposure variables were standardized (z-scores) | | |

| **Supplementary Table 7**. Associations between lean mass (kg), fat mass (kg) and bone mineral density (g/cm^2^) in different anatomical regions, by DII categories in **male** of the Health Workers Cohort Study. | | | | |
| --- | --- | --- | --- | --- |
|  | **DII categories**^a^ | | | **P value interaction** |
|  | **Low category**  **(n= 146)** | **Medium category**  **(n= 146)** | **High category**  **(n= 145)** |  |
|  | β (95% CI) | β (95% CI) | β (95% CI) |  |
| **Total BMD,** g/cm^2^ |  |  |  |  |
| Lean mass | 0.056 (0.035,0.076) | 0.049 (0.031,0.067) | 0.033 (0.017,0.050) | 0.3733 |
| Fat mass | -0.060 (-0.081,-0.039) | -0.052 (-0.071,-0.034) | -0.036 (-0.053,-0.019) | 0.4069 |
| Truncal fat mass | -0.001 (-0.011,0.009) | -0.012 (-0.027,0.004) | -0.008 (-0.023,0.006) | 0.8111 |
| Waist Circumference | -0.040 (-0.056,-0.024) | -0.028 (-0.041,-0.015) | -0.023 (-0.036,-0.009) | 0.0600 |
| **Sub-total BMD,** g/cm^2^ |  |  |  |  |
| Lean mass | 0.061 (0.043,0.080) | 0.062 (0.045,0.079) | 0.047 (0.032,0.063) | 0.7383 |
| Fat mass | -0.064 (-0.084,-0.045) | -0.065 (-0.082,-0.047) | -0.052 (-0.067,-0.036) | 0.7306 |
| Truncal fat mass | -0.003 (-0.013,0.007) | -0.013 (-0.028,0.003) | -0.014 (-0.029,-0.00008) | 0.5352 |
| Waist Circumference | -0.037 (-0.053,-0.022) | -0.026 (-0.039,-0.013) | -0.026 (-0.040,-0.013) | 0.2833 |
| **Lumbar Spine BMD,** g/cm^2^ |  |  |  |  |
| Lean mass | 0.055 (0.027,0.083) | 0.054 (0.031,0.077) | 0.039 (0.019,0.059) | 0.0417 |
| Fat mass | -0.069 (-0.097,-0.040) | -0.060 (-0.083,-0.037) | -0.046 (-0.066,-0.026) | 0.0335 |
| Truncal fat mass | -0.017 (-0.030,-0.003) | -0.004 (-0.023,0.015) | -0.005 (-0.023,0.013) | 0.0444 |
| Waist Circumference | -0.041 (-0.063,-0.018) | -0.025 (-0.041,-0.008) | -0.026 (-0.042,-0.009) | 0.0065 |
| **Hip BMD,** g/cm^2^ |  |  |  |  |
| Lean mass | 0.090 (0.057,0.124) | 0.089 (0.055,0.123) | 0.055 (0.023,0.086) | 0.6982 |
| Fat mass | -0.095 (-0.130,-0.060) | -0.095 (-0.129,-0.061) | -0.064 (-0.096,-0.032) | 0.5502 |
| Truncal fat mass | -0.001 (-0.018,0.015) | -0.012 (-0.041,0.017) | -0.020 (-0.048,0.007) | 0.4332 |
| Waist Circumference | -0.045 (-0.073,-0.017) | -0.036 (-0.061,-0.012) | -0.038 (-0.063,-0.012) | 0.9232 |
| **Arm BMD,** g/cm^2^ |  |  |  |  |
| Lean mass | 0.059 (0.039,0.080) | 0.051 (0.032,0.070) | 0.039 (0.021,0.057) | 0.8399 |
| Fat mass | -0.062 (-0.083,-0.040) | -0.052 (-0.071,-0.032) | -0.037 (-0.056,-0.018) | 0.8996 |
| Truncal fat mass | -0.004 (-0.014,0.007) | -0.009 (-0.025,0.007) | -0.012 (-0.028,0.004) | 0.8726 |
| Waist Circumference | -0.042 (-0.059,-0.026) | -0.028 (-0.041,-0.015) | -0.022 (-0.037,-0.008) | 0.2130 |
| **Leg BMD,** g/cm^2^ |  |  |  |  |
| Lean mass | 0.080 (0.054,0.106) | 0.086 (0.060,0.112) | 0.063 (0.039,0.087) | 0.9057 |
| Fat mass | -0.083 (-0.110,-0.056) | -0.090 (-0.116,-0.064) | -0.073 (-0.097,-0.048) | 0.4069 |
| Truncal fat mass | -0.019 (-0.041,0.003) | -0.023 (-0.046,0.0005) | -0.019 (-0.041,0.003) | 0.3421 |
| Waist Circumference | -0.044 (-0.066,-0.023) | -0.030 (-0.050,-0.010) | -0.033 (-0.054,-0.013) | 0.4271 |
| Models adjusted by age (years), physical activity leisure time (hours/week), calcium intake (EAR <1000, >1000 mg), smoking status (never, past and actual), tanner stage (five categorical stages: I to V) and weight(kg). ^a^DII categories defines by tertiles. Exposure variables were standardized (z-scores) | | | | |

| **Supplementary Table 8**. Associations between lean mass (kg), fat mass (kg) and bone mineral density (g/cm^2^) in different anatomical regions, by DII categories in **female** of the Health Workers Cohort Study. | | | | |
| --- | --- | --- | --- | --- |
|  | **DII categories**^a^ | | | **P value interaction** |
|  | **Low category**  **(n= 206)** | **Medium category**  **(n= 206)** | **High category**  **(n= 205)** |  |
|  | β (95% CI) | β (95% CI) | β (95% CI) |  |
| **Total BMD,** g/cm^2^ |  |  |  |  |
| Lean mass | 0.023 (-0.002,0.048) | 0.044 (0.020,0.067) | 0.040 (0.019,0.061) | 0.1283 |
| Fat mass | -0.029 (-0.054,-0.004) | -0.051 (-0.075,-0.028) | -0.041 (-0.063,-0.020) | 0.0714 |
| Truncal fat mass | -0.017 (-0.028,-0.006) | -0.00006 (-0.009,0.009) | 0.001 (-0.007,0.010) | 0.0055 |
| Waist Circumference | -0.015 (-0.026,-0.003) | -0.013 (-0.024,-0.003) | -0.014 (-0.023,-0.005) | 0.6081 |
| **Sub-total BMD,** g/cm^2^ |  |  |  |  |
| Lean mass | 0.034 (0.011,0.056) | 0.052 (0.031,0.073) | 0.050 (0.031,0.069) | 0.2775 |
| Fat mass | -0.050 (-0.069,-0.031) | -0.062 (-0.083,-0.042) | -0.037 (-0.059,-0.014) | 0.0994 |
| Truncal fat mass | -0.016 (-0.027,-0.006) | 0.002 (-0.006,0.010) | 0.0005 (-0.008,0.008) | 0.0022 |
| Waist Circumference | -0.015 (-0.026,-0.005) | -0.012 (-0.021,-0.002) | -0.014 (-0.022,-0.006) | 0.6393 |
| **Lumbar Spine BMD,** g/cm^2^ |  |  |  |  |
| Lean mass | 0.0003 (-0.034,0.034) | 0.031 (-0.005,0.067) | 0.029 (0.0003,0.058) | 0.3903 |
| Fat mass | -0.003 (-0.037,0.032) | -0.085 (-0.119,-0.051) | -0.046 (-0.075,-0.017) | 0.0075 |
| Truncal fat mass | -0.024 (-0.039,-0.009) | -0.006 (-0.019,0.006) | 0.003 (-0.009,0.014) | 0.0057 |
| Waist Circumference | -0.026 (-0.042,-0.010) | -0.021 (-0.035,-0.006) | -0.012 (-0.025,-0.0001) | 0.3333 |
| **Hip BMD,** g/cm^2^ |  |  |  |  |
| Lean mass | 0.038 (-0.004,0.080) | 0.064 (0.026,0.103) | 0.055 (0.019,0.091) | 0.4586 |
| Fat mass | -0.045 (-0.087,-0.003) | -0.076 (-0.114,-0.038) | -0.059 (-0.095,-0.023) | 0.2879 |
| Truncal fat mass | -0.005 (-0.025,0.014) | 0.014 (0.001,0.028) | 0.001 (-0.013,0.016) | 0.1737 |
| Waist Circumference | -0.016 (-0.036,0.004) | -0.010 (-0.027,0.006) | -0.013 (-0.028,0.003) | 0.4982 |
| **Arm BMD,** g/cm^2^ |  |  |  |  |
| Lean mass | 0.030 (0.008,0.052) | 0.037 (0.011,0.063) | 0.035 (0.009,0.061) | 0.6225 |
| Fat mass | -0.035 (-0.057,-0.013) | -0.046 (-0.072,-0.021) | -0.033 (-0.059,-0.006) | 0.2987 |
| Truncal fat mass | -0.008 (-0.018,0.002) | -0.018 (-0.027,-0.010) | -0.023 (-0.033,-0.013) | 0.2099 |
| Waist Circumference | -0.009 (-0.020,0.003) | -0.010 (-0.021,0.001) | -0.011 (-0.022,-0.0004) | 0.4325 |
| **Leg BMD,** g/cm^2^ |  |  |  |  |
| Lean mass | 0.060 (0.029,0.090) | 0.098 (0.069,0.127) | 0.074 (0.046,0.102) | 0.0501 |
| Fat mass | -0.063 (-0.094,-0.032) | -0.086 (-0.116,-0.057) | -0.074 (-0.102,-0.046) | 0.0714 |
| Truncal fat mass | -0.024 (-0.038,-0.010) | 0.004 (-0.007,0.015) | -0.012 (-0.023,0.0001) | 0.0058 |
| Waist Circumference | -0.021 (-0.036,-0.006) | -0.016 (-0.029,-0.003) | -0.022 (-0.035,-0.010) | 0.8695 |
| Models adjusted by age (years), physical activity leisure time (hours/week), calcium intake (EAR <1000, >1000 mg), smoking status (never, past and actual), tanner stage (five categorical stages: I to V) and weight(kg). ^a^DII categories defines by tertiles. Exposure variables were standardized (z-scores) | | | | |

| **Supplementary Table 9**. Associations between lean mass (kg), fat mass (kg) and bone mineral density (g/cm^2^) in different anatomical regions, by physical activity categories of the Health Workers Cohort Study. | | | | | | |
| --- | --- | --- | --- | --- | --- | --- |
|  | **Males** | | | **Females** | | |
|  | **PA <1 hour/day**  **(n=277)** | **PA >1 hour/day (n=160)** |  | **PA <1 hour/day (n=463)** | **PA >1 hour/day (n=154)** |  |
|  | **β (95% CI)** | **β (95% CI)** | **P value interaction** | **β (95% CI)** | **β (95% CI)** | **P value interaction** |
| **Total BMD,** g/cm^2^ |  |  |  |  |  |  |
| Lean mass | 0.036 (0.022,0.050) | 0.062 (0.043,0.082) | 0.550 | -0.0002 (-0.015,0.015) | 0.013 (-0.014,0.039) | 0.454 |
| Fat mass | -0.025 (-0.041,-0.010) | -0.068 (-0.088,-0.047) | 0.428 | -0.025 (-0.039,-0.010) | -0.043 (-0.071,-0.016) | 0.125 |
| Truncal fat mass | -0.000002 (-0.024,0.024) | -0.023 (-0.051,0.005) | 0.528 | -0.020 (-0.035,-0.005) | -0.029 (-0.062,0.004) | 0.479 |
| Waist Circumference | -0.021 (-0.031,-0.011) | -0.036 (-0.050,-0.021) | 0.825 | -0.014 (-0.021,-0.008) | -0.014 (-0.026,-0.001) | 0.728 |
| **Sub-total BMD,** g/cm^2^ |  |  |  |  |  |  |
| Lean mass | 0.034 (0.021,0.048) | 0.052 (0.032,0.072) | 0.476 | -0.006 (-0.019,0.008) | 0.013 (-0.010,0.036) | 0.345 |
| Fat mass | -0.037 (-0.049,-0.024) | -0.053 (-0.076,-0.030) | 0.040 | -0.044 (-0.058,-0.030) | -0.075 (-0.094,-0.056) | 0.404 |
| Truncal fat mass | -0.028 (-0.051,-0.004) | -0.042 (-0.068,-0.015) | 0.567 | -0.027 (-0.041,-0.014) | -0.039 (-0.067,-0.011) | 0.376 |
| Waist Circumference | -0.022 (-0.031,-0.012) | -0.030 (-0.045,-0.016) | 0.510 | -0.013 (-0.019,-0.007) | -0.016 (-0.026,-0.005) | 0.794 |
| **Lumbar Spine BMD,** g/cm^2^ |  |  |  |  |  |  |
| Lean mass | 0.032 (0.013,0.050) | 0.068 (0.040,0.096) | 0.629 | -0.040 (-0.061,-0.019) | -0.002 (-0.040,0.035) | 0.210 |
| Fat mass | -0.028 (-0.047,-0.009) | -0.080 (-0.109,-0.052) | 0.172 | -0.022 (-0.042,-0.001) | -0.081 (-0.118,-0.044) | 0.116 |
| Truncal fat mass | 0.008 (-0.023,0.038) | -0.056 (-0.093,-0.019) | 0.641 | -0.029 (-0.050,-0.007) | -0.068 (-0.113,-0.024) | 0.269 |
| Waist Circumference | -0.020 (-0.033,-0.008) | -0.032 (-0.053,-0.012) | 0.534 | -0.017 (-0.026,-0.008) | -0.029 (-0.046,-0.013) | 0.126 |
| **Hip BMD,** g/cm^2^ |  |  |  |  |  |  |
| Lean mass | 0.061 (0.036,0.087) | 0.101 (0.065,0.137) | 0.792 | 0.024 (-0.002,0.049) | 0.034 (-0.008,0.076) | 0.572 |
| Fat mass | -0.054 (-0.082,-0.027) | -0.109 (-0.146,-0.072) | 0.124 | -0.033 (-0.058,-0.008) | -0.052 (-0.097,-0.008) | 0.127 |
| Truncal fat mass | -0.012 (-0.056,0.031) | -0.035 (-0.086,0.015) | 0.922 | -0.002 (-0.028,0.024) | 0.004 (-0.048,0.057) | 0.195 |
| Waist Circumference | -0.030 (-0.048,-0.012) | -0.045 (-0.072,-0.018) | 0.375 | -0.017 (-0.028,-0.005) | -0.007 (-0.026,0.013) | 0.309 |
| **Arm BMD,** g/cm^2^ |  |  |  |  |  |  |
| Lean mass | 0.044 (0.028,0.059) | 0.053 (0.033,0.074) | 0.106 | 0.004 (-0.012,0.021) | 0.034 (0.011,0.057) | 0.333 |
| Fat mass | -0.031 (-0.047,-0.014) | -0.058 (-0.079,-0.037) | 0.166 | -0.025 (-0.041,-0.009) | -0.027 (-0.052,-0.002) | 0.460 |
| Truncal fat mass | -0.006 (-0.032,0.020) | -0.028 (-0.056,0.001) | 0.296 | -0.048 (-0.064,-0.032) | -0.001 (-0.031,0.029) | 0.964 |
| Waist Circumference | -0.023 (-0.034,-0.012) | -0.033 (-0.048,-0.018) | 0.816 | -0.010 (-0.017,-0.002) | -0.014 (-0.025,-0.003) | 0.517 |
| **Leg BMD,** g/cm^2^ |  |  |  |  |  |  |
| Lean mass | 0.034 (0.013,0.055) | 0.050 (0.020,0.080) | 0.340 | -0.011 (-0.030,0.009) | 0.005 (-0.027,0.038) | 0.344 |
| Fat mass | -0.069 (-0.090,-0.049) | -0.101 (-0.128,-0.073) | 0.023 | -0.063 (-0.081,-0.045) | -0.078 (-0.110,-0.045) | 0.815 |
| Truncal fat mass | -0.063 (-0.096,-0.029) | -0.067 (-0.106,-0.029) | 0.421 | -0.057 (-0.076,-0.038) | -0.071 (-0.109,-0.032) | 0.597 |
| Waist Circumference | -0.025 (-0.040,-0.011) | -0.034 (-0.055,-0.012) | 0.497 | -0.018 (-0.026,-0.010) | -0.022 (-0.037,-0.008) | 0.713 |
| Models adjusted by age (years), dietary inflammatory index (categories defined by tertiles), calcium intake (EAR <1000mg, >1000 mg), weight (kg), smoking status (never, past and actual) and tanner stage (five categorical stages: I to V). Exposure variables were standardized (z-scores) | | | | | | |

| **Supplementary Table 10**. Associations between lean mass (kg), fat mass (kg) and bone mineral density (g/cm^2^) in different anatomical regions, by calcium intake categories of the Health Workers Cohort Study. | | | | | | |
| --- | --- | --- | --- | --- | --- | --- |
|  | **Males** | | | **Females** | | |
|  | **EAR <1000 (n=215)** | **EAR >1000 (n=222)** |  | **EAR <1000 (n=375)** | **EAR >1000 (n=242)** |  |
|  | **β (95% CI)** | **β (95% CI)** | **P value interaction** | **β (95% CI)** | **β (95% CI)** | **P value interaction** |
| **Total BMD,** g/cm^2^ |  |  |  |  |  |  |
| Lean mass | 0.048 (0.030,0.066) | 0.040 (0.026,0.055) | 0.390 | 0.014 (-0.004,0.032) | -0.009 (-0.028,0.010) | 0.177 |
| Fat mass | -0.053 (-0.071,-0.035) | -0.032 (-0.048,-0.015) | 0.076 | -0.037 (-0.054,-0.020) | -0.018 (-0.037,0.001) | 0.739 |
| Truncal fat mass | -0.030 (-0.061,0.002) | -0.005 (-0.026,0.017) | 0.887 | -0.022 (-0.040,-0.004) | -0.021 (-0.042,0.0002) | 0.585 |
| Waist Circumference | -0.029 (-0.041,-0.017) | -0.024 (-0.035,-0.013) | 0.047 | -0.016 (-0.024,-0.009) | -0.009 (-0.018,-0.001) | 0.422 |
| **Sub-total BMD,** g/cm^2^ |  |  |  |  |  |  |
| Lean mass | 0.046 (0.028,0.063) | 0.033 (0.019,0.048) | 0.598 | 0.007 (-0.009,0.023) | -0.009 (-0.026,0.008) | 0.432 |
| Fat mass | -0.066 (-0.082,-0.049) | -0.047 (-0.063,-0.032) | 0.144 | -0.045 (-0.060,-0.031) | -0.033 (-0.050,-0.016) | 0.584 |
| Truncal fat mass | -0.051 (-0.081,-0.021) | -0.029 (-0.050,-0.008) | 0.743 | -0.028 (-0.044,-0.012) | -0.031 (-0.050,-0.012) | 0.660 |
| Waist Circumference | -0.029 (-0.041,-0.017) | -0.021 (-0.032,-0.010) | 0.113 | -0.016 (-0.023,-0.009) | -0.008 (-0.016,-0.0003) | 0.353 |
| **Lumbar Spine BMD,** g/cm^2^ |  |  |  |  |  |  |
| Lean mass | 0.043 (0.018,0.068) | 0.042 (0.024,0.060) | 0.030 | -0.020 (-0.044,0.005) | -0.041 (-0.068,-0.014) | 0.288 |
| Fat mass | -0.047 (-0.072,-0.021) | -0.052 (-0.071,-0.032) | 0.591 | -0.047 (-0.070,-0.024) | -0.011 (-0.039,0.017) | 0.391 |
| Truncal fat mass | -0.005 (-0.047,0.038) | -0.036 (-0.062,-0.011) | 0.018 | -0.037 (-0.062,-0.012) | -0.030 (-0.061,-0.0002) | 0.276 |
| Waist Circumference | -0.027 (-0.044,-0.011) | -0.022 (-0.036,-0.008) | 0.060 | -0.026 (-0.037,-0.016) | -0.008 (-0.020,0.004) | 0.345 |
| **Hip BMD,** g/cm^2^ |  |  |  |  |  |  |
| Lean mass | 0.075 (0.044,0.106) | 0.075 (0.048,0.103) | 0.798 | 0.037 (0.008,0.066) | 0.013 (-0.020,0.045) | 0.361 |
| Fat mass | -0.086 (-0.118,-0.054) | -0.069 (-0.099,-0.038) | 0.828 | -0.036 (-0.064,-0.008) | -0.041 (-0.074,-0.008) | 0.492 |
| Truncal fat mass | -0.046 (-0.100,0.009) | -0.015 (-0.055,0.026) | 0.910 | 0.005 (-0.025,0.036) | -0.011 (-0.047,0.025) | 0.659 |
| Waist Circumference | -0.034 (-0.056,-0.012) | -0.038 (-0.059,-0.017) | 0.822 | -0.015 (-0.028,-0.002) | -0.009 (-0.024,0.006) | 0.715 |
| **Arm BMD,** g/cm^2^ |  |  |  |  |  |  |
| Lean mass | 0.056 (0.038,0.075) | 0.034 (0.018,0.050) | 0.108 | 0.007 (-0.011,0.026) | 0.012 (-0.009,0.032) | 0.223 |
| Fat mass | -0.055 (-0.074,-0.036) | -0.030 (-0.047,-0.013) | 0.111 | -0.028 (-0.045,-0.010) | -0.019 (-0.040,0.001) | 0.160 |
| Truncal fat mass | -0.023 (-0.057,0.010) | -0.020 (-0.043,0.002) | 0.620 | -0.038 (-0.056,-0.019) | -0.044 (-0.066,-0.022) | 0.274 |
| Waist Circumference | -0.034 (-0.047,-0.022) | -0.020 (-0.032,-0.008) | 0.006 | -0.011 (-0.019,-0.003) | -0.008 (-0.017,0.001) | 0.445 |
| **Leg BMD,** g/cm^2^ |  |  |  |  |  |  |
| Lean mass | 0.045 (0.019,0.072) | 0.032 (0.010,0.054) | 0.767 | 0.006 (-0.017,0.028) | -0.018 (-0.043,0.007) | 0.427 |
| Fat mass | -0.097 (-0.121,-0.073) | -0.066 (-0.088,-0.043) | 0.259 | -0.072 (-0.092,-0.051) | -0.061 (-0.086,-0.037) | 0.334 |
| Truncal fat mass | -0.106 (-0.149,-0.063) | -0.046 (-0.076,-0.016) | 0.322 | -0.052 (-0.075,-0.030) | -0.074 (-0.100,-0.048) | 0.318 |
| Waist Circumference | -0.035 (-0.053,-0.018) | -0.022 (-0.038,-0.005) | 0.425 | -0.023 (-0.033,-0.013) | -0.011 (-0.022,0.0003) | 0.334 |
| Models adjusted by age (years), dietary inflammatory index (categories defined by tertiles), physical activity leisure time (hours/week), weight (kg), smoking status (never, past and actual) and tanner stage (five categorical stages: I to V). Exposure variables were standardized (z-scores) | | | | | | |

| **Supplementary Table 11.** Associations between lean mass (kg), fat mass (kg) and bone mineral density (g/cm2) in different anatomical regions, by weight categories of the Health Workers Cohort Study. | | | | | | |
| --- | --- | --- | --- | --- | --- | --- |
|  | **Males** | | | **Females** | | |
|  | **Low category (n=218)** | **High category (n=307)** |  | **Low category (n=310)** | **High category (n=307)** |  |
|  | **β (95% CI)** | **β (95% CI)** | **P value interaction** | **β (95% CI)** | **β (95% CI)** | **P value interaction** |
| **Total BMD,** g/cm^2^ |  |  |  |  |  |  |
| Lean mass | 0.036 (0.018,0.054) | 0.049 (0.033,0.066) | 0.791 | 0.005 (-0.015,0.024) | 0.013 (-0.005,0.031) | 0.149 |
| Fat mass | -0.038 (-0.056,-0.020) | -0.048 (-0.066,-0.030) | 0.098 | -0.023 (-0.042,-0.004) | -0.027 (-0.046,-0.008) | 0.319 |
| Truncal fat mass | -0.005 (-0.033,0.024) | -0.022 (-0.048,0.005) | 0.349 | -0.004 (-0.024,0.015) | -0.025 (-0.046,-0.004) | 0.012 |
| Waist Circumference | -0.025 (-0.036,-0.014) | -0.030 (-0.042,-0.017) | 0.024 | -0.016 (-0.024,-0.008) | -0.013 (-0.021,-0.005) | 0.459 |
| **Sub-total BMD,** g/cm^2^ |  |  |  |  |  |  |
| Lean mass | 0.033 (0.017,0.050) | 0.051 (0.036,0.067) | 0.013 | -0.002 (-0.019,0.015) | 0.013 (-0.003,0.029) | 0.078 |
| Fat mass | -0.054 (-0.070,-0.039) | -0.052 (-0.070,-0.035) | 0.265 | -0.040 (-0.056,-0.024) | -0.031 (-0.048,-0.014) | 0.148 |
| Truncal fat mass | -0.031 (-0.057,-0.006) | -0.028 (-0.054,-0.002) | 0.036 | -0.014 (-0.031,0.003) | -0.027 (-0.046,-0.008) | <0.001 |
| Waist Circumference | -0.030 (-0.040,-0.020) | -0.030 (-0.043,-0.018) | 0.499 | -0.016 (-0.023,-0.008) | -0.014 (-0.021,-0.007) | 0.762 |
| **Lumbar Spine BMD,** g/cm^2^ |  |  |  |  |  |  |
| Lean mass | 0.037 (0.017,0.057) | 0.044 (0.021,0.067) | 0.668 | -0.020 (-0.045,0.006) | -0.028 (-0.054,-0.002) | 0.051 |
| Fat mass | -0.041 (-0.061,-0.022) | -0.052 (-0.077,-0.027) | 0.004 | -0.024 (-0.049,0.001) | -0.036 (-0.064,-0.009) | 0.040 |
| Truncal fat mass | 0.006 (-0.025,0.037) | -0.042 (-0.078,-0.006) | 0.007 | 0.0004 (-0.026,0.027) | -0.059 (-0.089,-0.029) | <0.001 |
| Waist Circumference | -0.030 (-0.042,-0.018) | -0.026 (-0.044,-0.008) | 0.021 | -0.015 (-0.026,-0.004) | -0.026 (-0.038,-0.015) | 0.105 |
| **Hip BMD,** g/cm^2^ |  |  |  |  |  |  |
| Lean mass | 0.080 (0.049,0.111) | 0.083 (0.053,0.112) | 0.035 | 0.025 (-0.008,0.058) | 0.043 (0.013,0.073) | 0.189 |
| Fat mass | -0.083 (-0.114,-0.053) | -0.066 (-0.100,-0.033) | 0.627 | -0.041 (-0.073,-0.009) | -0.025 (-0.057,0.006) | 0.422 |
| Truncal fat mass | -0.016 (-0.066,0.034) | -0.012 (-0.061,0.036) | 0.127 | 0.0004 (-0.033,0.034) | 0.021 (-0.014,0.056) | 0.078 |
| Waist Circumference | -0.047 (-0.067,-0.028) | -0.035 (-0.059,-0.012) | 0.784 | -0.023 (-0.037,-0.009) | -0.008 (-0.022,0.005) | 0.243 |
| **Arm BMD,** g/cm^2^ |  |  |  |  |  |  |
| Lean mass | 0.040 (0.023,0.057) | 0.052 (0.034,0.070) | 0.334 | -0.0004 (-0.024,0.024) | 0.027 (0.010,0.043) | 0.942 |
| Fat mass | -0.036 (-0.054,-0.019) | -0.045 (-0.065,-0.025) | 0.143 | -0.018 (-0.041,0.005) | -0.024 (-0.042,-0.007) | 0.174 |
| Truncal fat mass | -0.0005 (-0.028,0.027) | -0.028 (-0.057,0.002) | 0.090 | -0.066 (-0.089,-0.043) | -0.009 (-0.029,0.011) | 0.206 |
| Waist Circumference | -0.022 (-0.033,-0.011) | -0.035 (-0.048,-0.021) | 0.017 | -0.012 (-0.022,-0.002) | -0.010 (-0.018,-0.003) | 0.918 |
| **Leg BMD,** g/cm^2^ |  |  |  |  |  |  |
| Lean mass | 0.035 (0.011,0.060) | 0.056 (0.032,0.079) | <0.001 | 0.007 (-0.016,0.030) | 0.008 (-0.015,0.032) | 0.001 |
| Fat mass | -0.074 (-0.097,-0.052) | -0.072 (-0.097,-0.047) | 0.176 | -0.058 (-0.079,-0.037) | -0.052 (-0.076,-0.029) | 0.003 |
| Truncal fat mass | -0.056 (-0.093,-0.019) | -0.047 (-0.084,-0.010) | 0.004 | -0.036 (-0.059,-0.014) | -0.052 (-0.078,-0.025) | <0.001 |
| Waist Circumference | -0.038 (-0.053,-0.023) | -0.038 (-0.056,-0.021) | 0.865 | -0.020 (-0.030,-0.011) | -0.021 (-0.032,-0.011) | 0.618 |
| Models adjusted by age (years), dietary inflammatory index (categories defined by tertiles), physical activity leisure time (hours/week), calcium intake (EAR <1000mg, >1000 mg), smoking status (never, past and actual) and tanner stage (five categorical stages: I to V). Exposure variables were standardized (z-scores). The low and high weight categories correspond to the median weight within each sex. | | | | | | |

| **Supplementary Table 12**. Associations between lean mass (kg), fat mass (kg) and bone mineral density (g/cm^2^) in different anatomical regions, by smoking status in **male** of the Health Workers Cohort Study. | | | | |
| --- | --- | --- | --- | --- |
|  | **Smoking status** | | | **P value interaction** |
|  | **Never(n=341)** | **Past(n=43)** | **Current(n=39)** |  |
|  | β (95% CI) | β (95% CI) | β (95% CI) |  |
| **Total BMD,** g/cm^2^ |  |  |  |  |
| Lean mass | 0.046 (0.033,0.059) | 0.030 (-0.014,0.073) | 0.054 (0.019,0.089) | 0.1214 |
| Fat mass | -0.042 (-0.054,-0.029) | -0.030 (-0.077,0.017) | -0.061 (-0.107,-0.016) | 0.6354 |
| Truncal fat mass | -0.019 (-0.040,0.003) | -0.034 (-0.114,0.047) | -0.016 (-0.064,0.032) | 0.4806 |
| Waist Circumference | -0.029 (-0.038,-0.021) | -0.011 (-0.049,0.026) | -0.012 (-0.043,0.020) | 0.2320 |
| **Sub-total BMD,** g/cm^2^ |  |  |  |  |
| Lean mass | 0.041 (0.028,0.054) | 0.033 (-0.006,0.073) | 0.057 (0.024,0.091) | 0.0494 |
| Fat mass | -0.057 (-0.069,-0.045) | -0.039 (-0.081,0.004) | -0.066 (-0.110,-0.022) | 0.6482 |
| Truncal fat mass | -0.046 (-0.067,-0.025) | -0.041 (-0.115,0.033) | -0.023 (-0.070,0.025) | 0.2756 |
| Waist Circumference | -0.028 (-0.037,-0.020) | -0.016 (-0.050,0.019) | -0.010 (-0.042,0.021) | 0.1609 |
| **Lumbar Spine BMD,** g/cm^2^ |  |  |  |  |
| Lean mass | 0.045 (0.028,0.062) | 0.035 (-0.016,0.086) | 0.077 (0.016,0.138) | 0.1638 |
| Fat mass | -0.050 (-0.066,-0.034) | -0.053 (-0.106,0.0002) | -0.074 (-0.154,0.007) | 0.1818 |
| Truncal fat mass | -0.014 (-0.041,0.013) | -0.073 (-0.165,0.019) | -0.057 (-0.135,0.021) | 0.0466 |
| Waist Circumference | -0.031 (-0.043,-0.020) | -0.038 (-0.081,0.005) | 0.011 (-0.041,0.063) | 0.1787 |
| **Hip BMD,** g/cm^2^ |  |  |  |  |
| Lean mass | 0.071 (0.048,0.095) | 0.074 (0.001,0.147) | 0.113 (0.045,0.182) | 0.2495 |
| Fat mass | -0.066 (-0.089,-0.043) | -0.083 (-0.161,-0.005) | -0.150 (-0.236,-0.064) | 0.1140 |
| Truncal fat mass | -0.027 (-0.065,0.011) | -0.073 (-0.212,0.065) | -0.023 (-0.120,0.073) | 0.3512 |
| Waist Circumference | -0.038 (-0.053,-0.022) | -0.022 (-0.087,0.043) | -0.015 (-0.078,0.048) | 0.5362 |
| **Arm BMD,** g/cm^2^ |  |  |  |  |
| Lean mass | 0.052 (0.038,0.066) | 0.024 (-0.019,0.068) | 0.059 (0.023,0.096) | 0.0117 |
| Fat mass | -0.045 (-0.058,-0.031) | -0.043 (-0.088,0.003) | -0.037 (-0.089,0.015) | 0.6230 |
| Truncal fat mass | -0.023 (-0.046,-0.0003) | -0.052 (-0.131,0.027) | -0.014 (-0.065,0.037) | 0.3172 |
| Waist Circumference | -0.032 (-0.041,-0.022) | -0.018 (-0.056,0.019) | -0.009 (-0.042,0.025) | 0.2114 |
| **Leg BMD,** g/cm^2^ |  |  |  |  |
| Lean mass | 0.041 (0.021,0.060) | 0.027 (-0.033,0.087) | 0.050 (-0.005,0.105) | 0.0807 |
| Fat mass | -0.082 (-0.100,-0.065) | -0.044 (-0.108,0.021) | -0.083 (-0.151,-0.016) | 0.9310 |
| Truncal fat mass | -0.085 (-0.114,-0.056) | -0.066 (-0.175,0.043) | -0.033 (-0.102,0.037) | 0.1624 |
| Waist Circumference | -0.032 (-0.044,-0.019) | -0.008 (-0.060,0.044) | -0.021 (-0.067,0.025) | 0.3153 |
| Models adjusted by age (years), physical activity leisure time (hours/week), calcium intake (EAR <1000, >1000 mg), tanner stage (five categorical stages: I to V) and weight(kg). ^a^DII categories defines by tertiles. Exposure variables were standardized (z-scores) | | | | |

| **Supplementary Table 13**. Associations between lean mass (kg), fat mass (kg) and bone mineral density (g/cm^2^) in different anatomical regions, by smoking status in **female** of the Health Workers Cohort Study. | | | | |
| --- | --- | --- | --- | --- |
|  | **Smoking status** | | | **P value interaction** |
|  | **Never (n=493)** | **Past(n=41)** | **Current(n=55)** |  |
|  | β (95% CI) | β (95% CI) | β (95% CI) |  |
| **Total BMD,** g/cm^2^ |  |  |  |  |
| Lean mass | -0.009 (-0.024,0.005) | 0.044 (-0.039,0.127) | 0.018 (-0.033,0.069) | 0.9456 |
| Fat mass | -0.038 (-0.052,-0.025) | -0.047 (-0.118,0.024) | -0.040 (-0.098,0.018) | 0.1920 |
| Truncal fat mass | -0.035 (-0.049,-0.020) | -0.022 (-0.125,0.081) | 0.024 (-0.041,0.090) | 0.1276 |
| Waist Circumference | -0.011 (-0.017,-0.004) | -0.044 (-0.070,-0.017) | -0.008 (-0.029,0.013) | 0.1287 |
| **Sub-total BMD,** g/cm^2^ |  |  |  |  |
| Lean mass | -0.014 (-0.027,-0.001) | 0.039 (-0.041,0.118) | 0.024 (-0.020,0.069) | 0.9467 |
| Fat mass | -0.052 (-0.064,-0.041) | -0.044 (-0.112,0.024) | -0.039 (-0.090,0.011) | 0.0920 |
| Truncal fat mass | -0.044 (-0.057,-0.031) | -0.021 (-0.119,0.077) | 0.028 (-0.029,0.086) | 0.2829 |
| Waist Circumference | -0.011 (-0.017,-0.005) | -0.036 (-0.062,-0.010) | -0.008 (-0.026,0.011) | 0.1136 |
| **Lumbar Spine BMD,** g/cm^2^ |  |  |  |  |
| Lean mass | -0.048 (-0.068,-0.029) | 0.003 (-0.107,0.113) | 0.003 (-0.071,0.078) | 0.8383 |
| Fat mass | -0.052 (-0.071,-0.033) | -0.031 (-0.125,0.063) | -0.026 (-0.111,0.059) | 0.2670 |
| Truncal fat mass | -0.055 (-0.075,-0.035) | -0.058 (-0.190,0.074) | 0.021 (-0.074,0.117) | 0.4711 |
| Waist Circumference | -0.016 (-0.025,-0.007) | -0.055 (-0.091,-0.020) | -0.017 (-0.047,0.012) | 0.1866 |
| **Hip BMD,** g/cm^2^ |  |  |  |  |
| Lean mass | 0.008 (-0.015,0.031) | 0.031 (-0.097,0.159) | 0.087 (-0.006,0.179) | 0.6430 |
| Fat mass | -0.055 (-0.077,-0.032) | -0.003 (-0.114,0.108) | -0.049 (-0.158,0.061) | 0.7739 |
| Truncal fat mass | -0.024 (-0.048,0.0003) | 0.072 (-0.082,0.226) | 0.113 (-0.007,0.232) | 0.9873 |
| Waist Circumference | -0.011 (-0.021,0.0002) | -0.054 (-0.098,-0.011) | 0.018 (-0.021,0.057) | 0.0412 |
| **Arm BMD,** g/cm^2^ |  |  |  |  |
| Lean mass | -0.001 (-0.016,0.014) | 0.047 (-0.051,0.146) | 0.019 (-0.026,0.064) | 0.6281 |
| Fat mass | -0.039 (-0.053,-0.025) | -0.069 (-0.151,0.014) | -0.006 (-0.058,0.047) | 0.0451 |
| Truncal fat mass | -0.057 (-0.072,-0.042) | -0.036 (-0.156,0.085) | 0.023 (-0.035,0.082) | 0.1963 |
| Waist Circumference | -0.009 (-0.016,-0.002) | -0.033 (-0.068,0.001) | 0.001 (-0.018,0.020) | 0.2509 |
| **Leg BMD,** g/cm^2^ |  |  |  |  |
| Lean mass | 0.032 (-0.029,0.092) | 0.008 (-0.090,0.106) | -0.023 (-0.042,-0.005) | 0.4658 |
| Fat mass | -0.083 (-0.100,-0.066) | -0.041 (-0.124,0.042) | -0.082 (-0.148,-0.016) | 0.0127 |
| Truncal fat mass | -0.079 (-0.098,-0.061) | -0.039 (-0.157,0.079) | 0.017 (-0.062,0.095) | 0.0674 |
| Waist Circumference | -0.017 (-0.026,-0.009) | -0.028 (-0.061,0.006) | -0.010 (-0.036,0.015) | 0.4676 |
| Models adjusted by age (years), physical activity leisure time (hours/week), calcium intake (EAR <1000, >1000 mg), tanner stage (five categorical stages: I to V) and weight(kg). ^a^DII categories defines by tertiles. Exposure variables were standardized (z-scores) | | | | |

| **Supplementary Table 14.** Multicollinearity Diagnostics: Variance Inflation Factors (VIF) for Exposure Variables in Linear Regression Models Stratified by Sex | | |
| --- | --- | --- |
|  | **Male** | **Female** |
| Lean mass | 2.30 | 1.76 |
| Fat mass | 2.83 | 2.56 |
| Truncal fat mass | 3.84 | 2.70 |
| Waist Circumference | 2.01 | 1.59 |
| Models adjusted by age (years), dietary inflammatory index (categories defined by tertiles), physical activity leisure time (hours/week), calcium intake (EAR <1000 mg, >1000 mg), weight (kg), smoking status (never, past and actual) and tanner stage (five categorical stages: I to V). Exposure variables were standardized (z-scores). Variance inflation factor (VIF): A VIF cutoff of < 5 was used to indicate the absence of significant multicollinearity among covariates. | | |
